# Nanoscale Structural Insights into Thermochromic VO_2_ Thin Films Using Tip-Enhanced Raman Spectroscopy

**DOI:** 10.1021/acsami.5c04827

**Published:** 2025-05-22

**Authors:** Ayushi Rai, Siiri Bienz, Vidar F. Hansen, Renato Zenobi, Naresh Kumar

**Affiliations:** † Department of Mechanical and Structural Engineering and Materials Science, 56627 University of Stavanger, N-4036 Stavanger, Norway; ‡ Department of Chemistry and Applied Biosciences, 27219 ETH Zurich, Vladimir-Prelog-Weg 1−5/10, 8093 Zurich, Switzerland

**Keywords:** thermochromic VO_2_ thin film, VO_2_/TiO_2_ bilayer, interfacial analysis, tip-enhanced Raman spectroscopy, nanoscale resolution, hyperspectral imaging

## Abstract

Thermochromic vanadium dioxide (VO_2_) thin films, known for their reversible metal–insulator transition (MIT) near 68 °C, are promising candidates for energy-efficient applications such as smart window coatings. However, optimizing their structural and interfacial properties to enhance thermochromic performance remains a significant challenge. Traditional characterization techniques such as X-ray diffraction and transmission electron microscopy face inherent limitations in simultaneously providing detailed chemical information and nanoscale spatial resolution—capabilities that are essential for resolving localized structural heterogeneity and interfacial phenomena. This study employs hyperspectral tip-enhanced Raman spectroscopy (TERS) imaging to address these limitations and investigate the nanoscale structure of pristine VO_2_ and VO_2_/TiO_2_ thin films. TERS imaging revealed nanoscale regions of lattice deformations and nanocrystallites with different orientations in VO_2_ thin films, resulting in a high density of grain boundaries that elevate the MIT temperature. In VO_2_/TiO_2_ bilayers, TERS detected coexisting anatase and brookite phases in the TiO_2_ layer, with tensile strain in the brookite phase and the VO_2_/TiO_2_ interface characterized by localized intermixing and strain. These novel insights underscore the polycrystalline nature of the thin films grown with pulsed layer deposition technique and highlight the critical role of nanoscale structural and interfacial properties in determining thermochromic performance of VO_2_-based thin films. Furthermore, this study demonstrates the effectiveness of TERS as a robust nanoanalytical tool for advancing the design of VO_2_-based smart coatings and functional materials.

## Graphical abstract

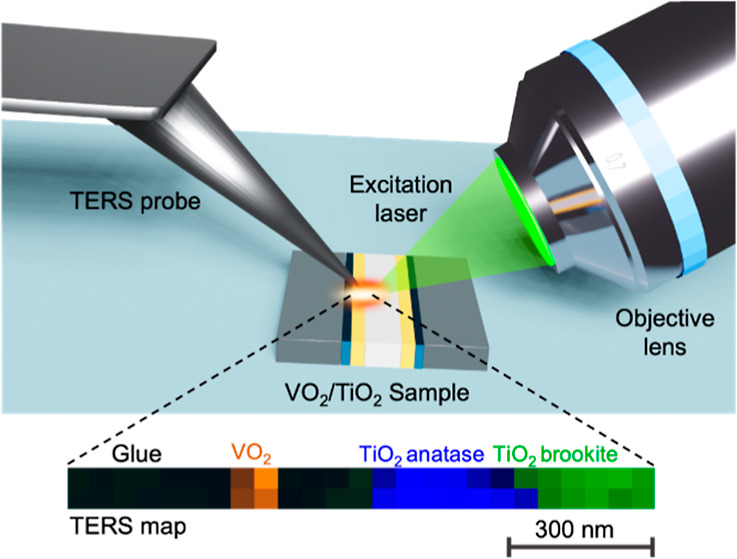

## 1 Introduction

In recent years, the development of advanced materials for energy-saving and clean energy applications has gained significant attention, driven by the pressing need for sustainable solutions to combat global warming.^1^ The increasing frequency of extreme weather events, rising global temperatures, and the urban heat island effect, particularly in densely populated regions, have escalated the demand for energy-efficient technologies.^2,3^ Among these, thermochromic coatings for smart windows have emerged as a promising solution, offering dynamic control of light and heat transmission to reduce energy consumption. Metal oxide thin films have been extensively explored in nanotechnology and materials science due to their versatile properties and wide-ranging applications in the electrochromic, photochromic, and thermochromic systems.^4^ For instance, the tunable nature of thin film properties, achieved through methods such as strain engineering and doping, enhances their adaptability across various semiconductor devices.^5^ In this context, vanadium dioxide (VO_2_) stands out as a material of particular interest due to its unique thermochromic properties, first extensively studied in the mid-20th century.^6,7^ VO_2_ undergoes a reversible metal-to-insulator transition (MIT) at approximately 68 °C, characterized by a phase change from a monoclinic M1 phase to a tetragonal rutile (R) phase. This transition is accompanied by a significant change in electrical conductivity and infrared reflectivity, making VO_2_ highly suitable for applications in sensors, smart windows, and other optical and electronic devices. Generally, inorganic thermochromic materials are promising for smart window applications due to their temperature-responsive optical properties. However, several candidates face critical limitations. For instance, CuHgI_4_ is toxic due to presence of mercury.^8^ V_2_O_5_ undergoes color changes upon heating, often irreversibly due to structural degradation, and offers minimal IR modulation.^9^ CuI shows a red-to-white transition via phase change, but the process may be structurally irreversible and lacks significant IR modulation.^10^ Some perovskite compounds have also been investigated, yet they commonly suffer from poor environmental stability under humidity and UV exposure, along with weak thermochromic contrast and limited IR regulation.^11^ In contrast, VO_2_, in addition to lacking visible color change, exhibits strong and reversible IR modulation and superior environmental robustness, making it a leading candidate for smart thermal regulation.^12^ To enhance the practicality of VO_2_ for smart window applications, strategies to lower the MIT temperature have been explored, with strain engineering emerging as a particularly effective approach. Introducing strain via lattice mismatch with a titanium dioxide (TiO_2_) thin film has been shown to successfully reduce the MIT temperature, thereby improving the performance of VO_2_-based thermochromic coatings.^13^

Structural characterization of VO_2_ thin films is traditionally performed using techniques such as X-ray diffraction (XRD) and transmission electron microscopy (TEM).^14^ However, these methods face certain limitations. XRD often struggles to distinguish between different VO_2_ polymorphs due to overlapping diffraction peaks, while TEM, despite offering high-resolution imaging, involves complex and time-consuming sample preparation. The requirement for ultrathin, undamaged samples using techniques like mechanical polishing or focused ion beam milling further complicates TEM analysis, with the high-energy electron beams potentially damaging VO_2_ film during imaging.^15,16^

Tip-enhanced Raman spectroscopy (TERS) has emerged as a powerful nanoanalytical tool for the investigation of thin films, overcoming the diffraction limit of conventional optical techniques and enabling material characterization with nanoscale spatial resolution.^17^ By positioning a metal-coated scanning probe microscopy tip at the focal spot of a Raman excitation laser, TERS utilizes localized surface plasmon resonance to enhance the electromagnetic field at the tip apex.^18,19^ This localized “near-field” enhancement facilitates spectroscopic measurements with nanoscale spatial resolution, high chemical sensitivity and molecular specificity.^20^ While Raman spectroscopy has been widely used to study VO_2_ thin films,^21–23^ to the best of our knowledge, nanoscale mapping of polycrystalline VO_2_ thin films using TERS has not been reported yet.

In this study, we employ hyperspectral TERS imaging to investigate the nanoscale structural characteristics of VO_2_ and VO_2_/TiO_2_ thin films for the first time. The TiO_2_ thin film serves as a strain-inducing layer to lower the MIT temperature of the VO_2_ film.^24^ Hyperspectral TERS imaging revealed a homogeneously distributed M1 phase and nanoscale regions of differing crystallite orientations within the pristine VO_2_ thin film. Notably, in the VO_2_/TiO_2_ thin-film sample, two different phases, anatase and brookite were detected in the TiO_2_ layer with 10 nm spatial resolution. Furthermore, the VO_2_/TiO_2_ interface exhibited intermixing at the nanoscale, rather than a sharp boundary between the two layers. These findings highlight the capability of hyperspectral TERS imaging for nanoscale structural investigation of VO_2_ and VO_2_/TiO_2_ thin films, enabling the identification of crystallite orientations, impurity phases, and interfacial intermixing—factors that directly influence the thermochromic properties of these thin films.

## 2 Experimental Methods

### 2.1 TERS System

TERS measurements were performed using a side-illumination NanoRaman system, as schematically depicted in Figure 1a. The integrated system combined a LabRam Soleil Raman spectrometer (HORIBA Scientific, France) with an atomic force microscope (AFM, HORIBA Scientific, France). Due to the opacity of the thin-film sample, a reflection mode TERS geometry was utilized. A 532 nm excitation laser was used, directed onto the sample surface at a 65° angle relative to the normal axis. The laser beam was focused at the TERS tip apex using a 100× objective lens with a numerical aperture of 0.7. TERS mapping was conducted using a laser power of 263 μW in the far-field laser spot at the sample and a spectral acquisition time of 20 s. An 1800 lines/mm grating with a spectral resolution of 2.4 cm^–1^ was employed. Instrumental drift of the TERS system was evaluated by performing AFM topography imaging of a Si calibration grid. Notably, drift was primarily observed between sets of 20 consecutive images, with an average displacement of 2.2 ± 1.1 nm between successive images. In contrast, negligible drift was detected during individual AFM scans of 35 min under experimental conditions (i.e., during excitation laser irradiation), and no distortion of the grid features was observed when imaged at a pixel size of 1.5 nm, which corresponds to a spatial resolution of ca. 3.5 nm according to the Nyquist criterion. Therefore, during TERS mapping, we expect the sample drift to remain <6 nm/h, although consecutive TERS images of the same area may exhibit spatial shifts of approximately 2.2 ± 1.1 nm.

**Figure 1:**
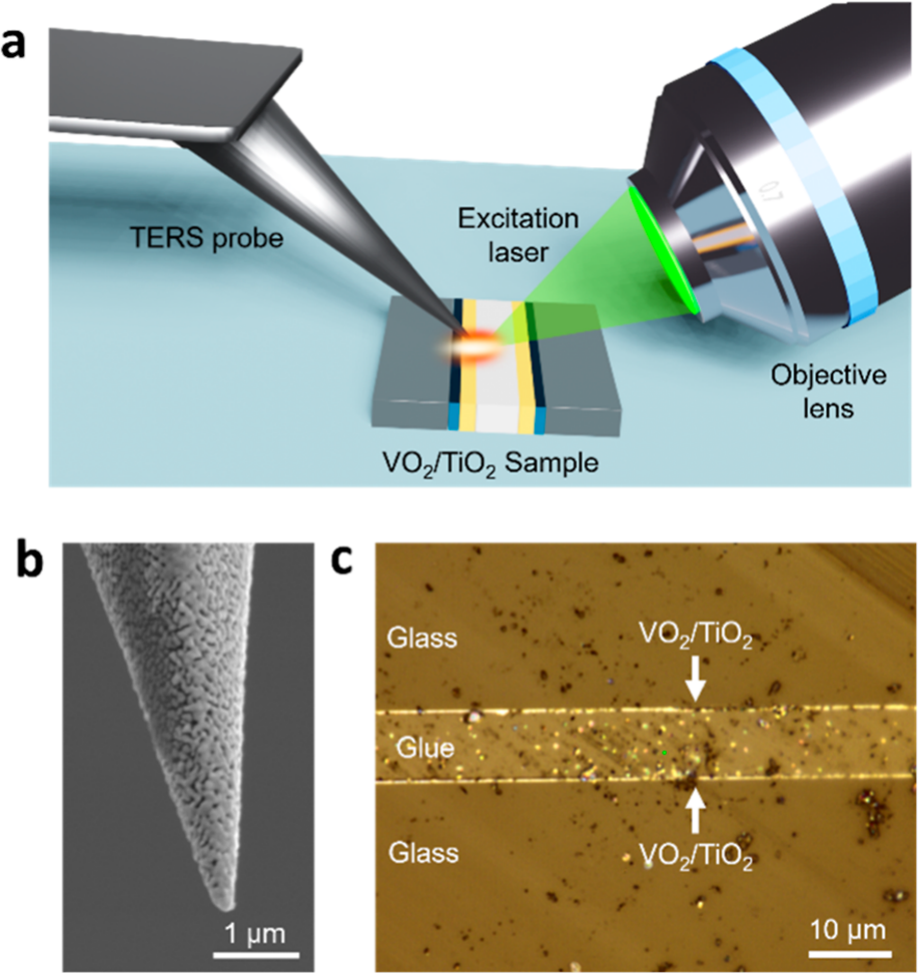
(a) Schematic representation of the side-illumination AFM-TERS setup used for the nanoscale investigation of thermochromic thin films in this study. Sample structure: glue (white), VO_2_ (orange), TiO_2_ (blue), and glass (gray). (b) SEM image of a representative Ag coated TERS tip used in this work. (c) Optical image showing cross-section of the VO_2_/TiO_2_/glass bilayer thin-film sample.

### 2.2 Preparation of TERS Probes

TERS probes were fabricated by first oxidizing Si AFM cantilevers in a high-temperature furnace (Carbolite Gero, UK) at 1000 °C for 23 h, a step necessary to decrease the surface refractive index. Subsequently, the cantilevers were subjected to UV–ozone cleaning (Ossila, UK) for 1 h to remove organic contaminants. The cleaned cantilevers were then transferred to a N_2_ glovebox (MBraun, Germany) equipped with a thermal evaporation system. Inside the glovebox, a 120 nm thick layer of high-purity Ag (99.99%, Advent Research Materials, UK) was deposited onto the cantilevers at a controlled deposition rate of 0.05 nm/s under ultrahigh vacuum conditions (<10^–6^ mbar). The evaporation source was oriented at 90° relative to the rotation axis, while the probes were mounted at an angle of 65°, with their tips aligned parallel to the axis of rotation. Throughout the evaporation process, the probes were continuously rotated around this axis to ensure uniform coating. A scanning electron microscopy (SEM) image of a representative Ag-coated tip is shown in Figure 1b. The typical apex diameter of the TERS tips was 86 ± 10 nm. To prevent oxidation and contamination, the Ag-coated probes were stored within the N_2_ glovebox, where the O_2_ and H_2_O levels were maintained below 0.1 ppm. TERS probes prepared by this method are capable of achieving a spatial resolution of at least 10 nm, as demonstrated in our previous study on Sb_2_Se_3_-based thin-film solar cells, where distinct chemical compounds were resolved within individual pixels of a TERS map acquired with a 10 nm step size.^17^ To exclude any SERS activity originating from the tip itself, tip spectra were checked before and after the TERS measurements.

### 2.3 Sample Preparation

VO_2_ thin films were fabricated using pulsed laser deposition (PLD), a method chosen for its precise control over deposition parameters, including oxygen partial pressure and substrate temperature, which are critical for achieving high crystalline quality.^25,26^ Thin films of VO_2_ and VO_2_/TiO_2_ were deposited on glass substrates, with the specific PLD parameters detailed in Table 1. For TERS analysis, cross-sectional samples were prepared by cutting the thin films with a microsaw and gluing them together to expose the internal layer structure, as shown in Figure 1c. The samples were polished using a MultiPrep system to achieve a flat surface with low roughness. VO_2_, being a brittle material, required careful handling during polishing. The MultiPrep system, designed specifically for delicate SEM and TEM sample preparation, was employed to progressively polish the samples using lapping films with grid sizes ranging from 1 to 0.1 μm. To mitigate temperature-induced alterations in the film during polishing, the process was carried out with a continuous flow of cold water over the polishing stage. Additionally, to minimize mechanical damage, the samples were polished with a consistent polishing direction maintained throughout the procedure. Note that, unlike TEM, TERS does not require extensive sample thinning, thereby allowing better preservation of the sample’s native structure. The RMS roughness of the VO_2_ monolayer and VO_2_/TiO_2_ bilayer samples was determined from the AFM topography images to be 7.6 ± 2.2 nm and 14.8 ± 3.3 nm, respectively. The increase in the bilayer roughness is attributed to the presence of an additional VO_2_/TiO_2_ interface, which introduces greater surface variation. The film thickness is expected to be on the order of several hundred nanometers. However, the surface roughness hindered the clear identification of the film–substrate interface, making it challenging to accurately determine the film thickness using AFM imaging.

**Table 1:** Parameters for PLD of VO_2_ and VO_2_/TiO_2_ Thin Films Investigated in This Study

	O_2_ partial pressure (Pa)		deposition temperature (°C)		no. of pulses	
sample	VO_2_	TiO_2_	VO_2_	TiO_2_	VO_2_	TiO_2_
VO_2_ thin film	0.8		600		5000	
VO_2_/TiO_2_ thin film	1	10	600	600	10,000	2000

### 2.4 Data Analysis

TERS spectra were processed using LabSpec 6 software (HORIBA Scientific, France), which included spike removal, baseline correction through polynomial fitting, and smoothing with a Savitzky–Golay filter. Spectral visualization and further analysis were performed using OriginPro 2021 (version 9.8.0.200). TERS maps were constructed based on the intensity (height) of Raman peaks obtained from the baseline-corrected spectra, without subtracting the far-field contribution. The peak intensities corresponding to the VO_2_ M1, TiO_2_ brookite, and TiO_2_ anatase phases were determined by integrating the TERS signals within the spectral ranges of 212–232 cm^–1^, 106–126 cm^–1^, and 122–170 cm^–1^, respectively. TERS maps of the peak positions were generated by extracting the spectral region corresponding to the peaks and fitting them with a Gaussian function. To analyze the peak positions of VO_2_ and TiO_2_ in the spatial maps (Figures 5d,e and S7d,e), a signal-to-noise (S/N) threshold of ≥5 was applied. Spectral data points with S/N values below this threshold were excluded from the peak position analysis and assigned a value of zero, as they were deemed unreliable for accurate spectral interpretation. For enhanced visualization, composite overlay images of the TERS maps were created by merging the individual phase-specific maps using the overlay functionality in Photoshop (version 25.11.0).

## 3 Results and Discussion

### 3.1 Nanoscale Structural Investigation of VO_2_ Thin Film

To confirm the plasmonic sensitivity of the Ag-coated TERS probes, we first compared far-field Raman spectra with TERS spectra acquired at similar locations on VO_2_ and VO_2_/TiO_2_ thin-film samples. As shown in Figure S1, the TERS signals exhibited up to 5-fold enhancement over the far-field Raman signals, highlighting the strong plasmonic performance of the probes. This signal enhancement resulting from the TERS near-field is critical for nanoscale analysis, particularly for VO_2_ thin films, where the typical crystallite size has been reported to be ∼30 nm.^27,28^ We performed TERS mapping across a cross-section of the VO_2_ thin film with a step size of 10 nm to assess its nanoscale structural characteristics. Figure 2a,b display TERS maps for the 222 cm^–1^ and 687 cm^–1^ Raman signals, respectively, with an overlay of these maps shown in Figure 2c. Notably, the 687 cm^–1^ signal exhibited greater variation across the thin film compared to the 222 cm^–1^ signal, signifying localized structural or compositional heterogeneity.

**Figure 2:**
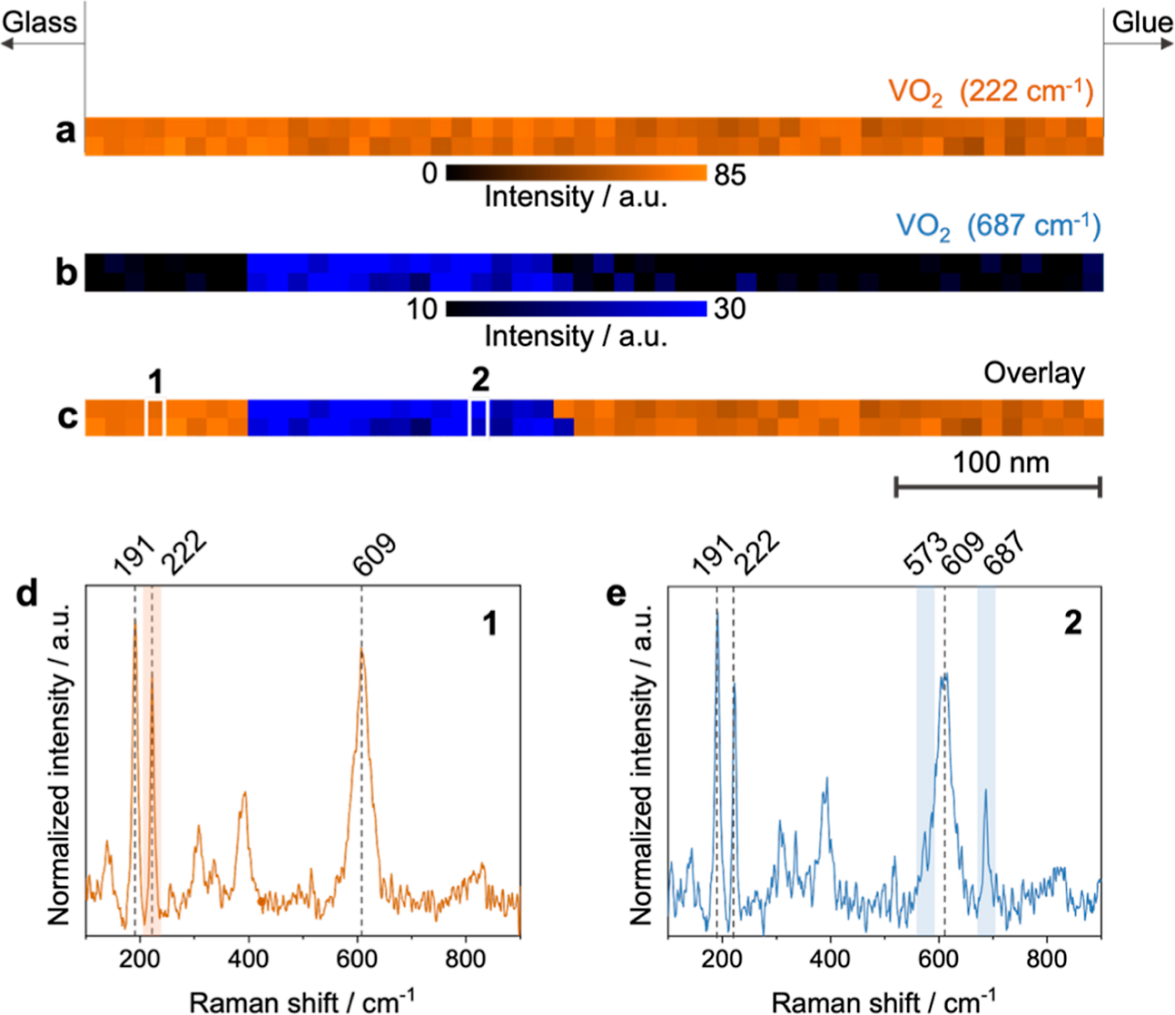
TERS maps of the VO_2_ thin film on a glass substrate showing the intensity distribution of (a) the 222 cm^–1^ and (b) the 687 cm^–1^ Raman peaks. Step size: 10 nm. Intensity of the 687 cm^–1^ peak was determined by integrating the 674–698 cm^–1^ spectral range. In the measured thin-film region, the glue and glass are on the right and left sides, respectively. (c) Overlay of the TERS maps from panels a and b, highlighting a region (blue) of approximately 150 nm within the VO_2_ thin film with a pronounced 687 cm^–1^ signal. Raman spectra (averaged over two pixels) from the locations marked as (d) 1 and (e) 2 in panel c. In (e), the two additional bands at 573 cm^–1^ and 687 cm^–1^ are marked in blue.

TERS spectra from specific regions marked as 1 and 2 in Figure 2c are shown in Figure 2d,e, respectively. In both spectra, peaks at 191 cm^–1^, 222 cm^–1^, 609 cm^–1^, and 822 cm^–1^ confirmed the presence of the VO_2_ M1 phase.^22,29,30^ A detailed assignment of the Raman peaks observed in the TERS spectra of VO_2_ thin film is provided in Figure S2 and Table S1. Intriguingly, the blue region in the overlay map (Figure 2c) revealed additional peaks at 573 cm^–1^ and 687 cm^–1^, highlighted in the average spectrum from this region (Figure 2e). VO_2_ is susceptible to oxidation and can exist in multiple oxidation states, with V–O vibrations typically appearing within the 250–700 cm^–1^ spectral range.^31^ However, the absence of any spectral changes below 573 cm^–1^ in the TERS spectra from location 2 strongly suggests that a distinct V_*X*_O_*Y*_ compound, such as V_2_O_5_, is not present at this site. In a previous study, Olivia Avilés et al. reported a TERS investigation of VS_2_ and VO_2_ and observed laser-induced conversion of VO_2_ into various VO_2_ polymorphs and V_2_O_5_.^32^ However, the Raman band positions in our TERS spectra did not match with those reported for the VO_2_/V_2_O_5_ mixture in that study, suggesting that such phase transformations did not occur under our experimental conditions. We attribute this to the significantly lower laser power used in our study. Specifically, the effective laser power at the tip apex in our measurements was approximately 2.3 μW, assuming a tip diameter of ∼86 nm (typical for our probes), which is roughly one-tenth of the laser power employed by Olivia Avilés et al. This low power greatly reduces the likelihood of thermally induced oxidation or phase transitions of the VO_2_ sample in the TERS near-field.

An alternative explanation could be the presence of the VO_2_ M2 phase within the thin film.^33–35^ Raman spectrum of the VO_2_ M2 phase is characterized by red-shifted peaks at 191 cm^–1^, 222 cm^–1^, and 609 cm^–1^ relative to the M1 phase. Since these shifts were absent in our TERS spectra, we conclude that there is no definitive evidence confirming the presence of the M2 phase in the VO_2_ thin film.

Previous studies have demonstrated that lattice deformations, doping, and thermal effects in VO_2_ nanocrystals can induce spectral modifications, including the emergence of new peaks and blue-shifting of V–O stretching modes within the 500–700 cm^–1^ range.^36^ Given that no doping was introduced in the VO_2_ thin film and the TERS measurements were conducted using a low laser power of 263 μW, the influence of doping and thermal expansion is expected to be negligible. Accordingly, we attribute the peaks observed at 573 cm^–1^ and 687 cm^–1^ in the blue region of Figure 2c to the strain caused by local lattice deformations affecting the V–O bonds in the VO_2_ thin film.

In the Raman spectrum of VO_2_ M1 phase, the 191 cm^–1^ Ag mode is associated with the vibration of V atoms along the [100] direction, while the 222 cm^–1^ mode represents vibrations of V atoms nearly perpendicular to this direction.^37^ Therefore, the intensity ratio of the 222 cm^–1^ and 191 cm^–1^ peaks (*I*_222_/*I*_191_) serves as a reliable metric for evaluating the crystallite orientation in VO_2_ thin films.^38^ Figure 3a presents a spatial map of the *I*_222_/*I*_191_ intensity ratio corresponding to the same VO_2_ thin-film region shown in Figure 2a. A notable variation in the *I*_222_/*I*_191_ intensity ratio, ranging from 0.5 (black regions) to 1 (blue regions), is evident at the nanoscale across the map. This variation, as highlighted by the representative spectra in Figure 3b, suggests the presence of nanocrystallites with different orientations within the VO_2_ thin film. In previous studies, the intensity of 338 cm^–1^ Raman signal has also been reported to be orientation-dependent.^38^ However, we did not observe it, presumably due to the weak 338 cm^–1^ signal and high noise level in our TERS measurements. Nonetheless, *I*_222_/*I*_191_ ratio analysis indicates the formation of numerous grain boundaries in the VO_2_ thin film, attributed to the small size and different orientations of the nanocrystallites. VO_2_ films with a reduced density of grain boundaries (i.e., larger grain size) have been shown to exhibit a comparatively lower MIT temperature (M1 → R phase).^39^ Therefore, the presence of nanocrystallites with different orientations, as shown in Figure 3a, is expected to increase the MIT temperature and negatively impact the optical and electronic properties of the VO_2_ thin film.

**Figure 3:**
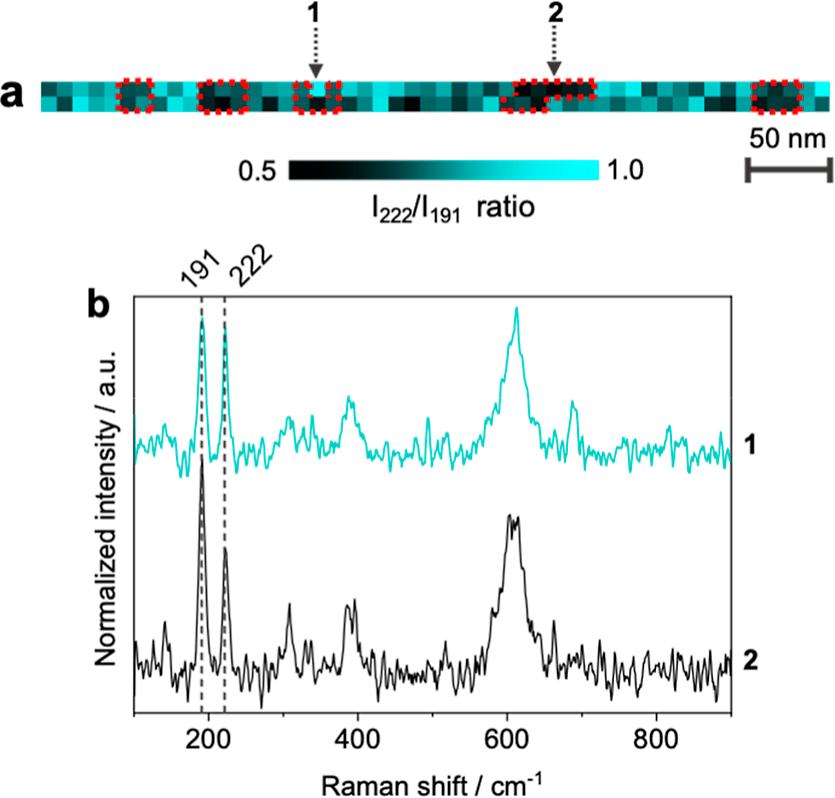
(a) TERS map of the *I*_222_/*I*_191_ ratio across the VO_2_ thin-film region shown in Figure 2a, revealing significant spatial variation with values ranging from 0.5 to 1. (b) TERS spectra obtained from the locations marked as 1 and 2 in panel a, normalized to the intensity of the 222 cm^–1^ peak for comparison.

Notably, the variable enhancement of Raman peaks in VO_2_ has indeed been reported previously and is known to depend on factors such as polarization and crystal orientation.^37^ In our case, we speculate that the observed variability in peak enhancement may be related to the symmetry of vibrational modes, as certain bonds in VO_2_ appear to be preferentially enhanced. However, interpreting these differences in terms of bond-specific enhancement is complicated by the polycrystalline nature of the thin film, which contains crystallites with several orientations. Furthermore, we also observed variation in the relative intensities of Raman peaks across different TERS tips. We attribute this tip-to-tip variability to slight differences in the localized surface plasmon resonance (LSPR) properties of the tips, which arise from the inherent stochasticity of the physical vapor deposition process used to fabricate them.

Under laser irradiation, oxidation of VO_2_ to V_*X*_O_*Y*_ has been reported in thin films.^31^ To investigate whether 532 nm laser irradiation induces oxidation-related spectral features in the Raman spectrum of VO_2_ thin films, we conducted control measurements under varying laser power and integration times, which are presented in Figures S3 and S4. The far-field Raman spectra revealed a transition from the VO_2_ M1 phase to the R phase (MIT) as the laser power was gradually increased from 0.18 mW to 3.20 mW (Figure S3). This phase transition initiated at 2.10 mW, with the 191 cm^–1^ peak undergoing a blue shift and the 609 cm^–1^ peak, associated with V–O stretching, exhibiting significant broadening. These spectral changes were fully reversed when the laser power was reduced back to 0.18 mW, signifying the reversibility of phase transition. In contrast, extending the integration time from 5 to 300 s produced no spectral changes in the far-field Raman spectra, confirming that prolonged laser exposure neither triggered phase transitions nor induced oxidation in the VO_2_ thin film (Figure S4). Note that only the transition from VO_2_ M1 to VO_2_ R phases is reversible, whereas the oxidation process from VO_2_ to V_*X*_O_*Y*_ is irreversible.

It is worth noting that while far-field Raman measurements confirm that increasing the laser power induces the expected phase transition in VO_2_ rather than oxidation of the film, no evidence of the VO_2_ (M1) to VO_2_ (R) phase transition was observed during our TERS measurements. This indicates that the local temperature in the TERS near-field at the sample remained below the transition threshold of 68 °C. Moreover, no additional thermally induced spectral features, such as increased background signal or baseline shifts, were observed during the TERS experiments. Such features would be expected if significant plasmonic heating were occurring. The absence of these changes supports the conclusion that thermal effects were minimal under our experimental conditions. Importantly, we reiterate that a very low laser power was used for the TERS measurements—263 μW in the far-field spot (corresponding to a power density of 390 μW/μm^2^), translating to an estimated effective power of approximately 2.3 μW at the tip apex. In prior studies, even with a total incident power of 60 μW in the far-field laser spot, the temperature rise in the TERS near-field has been shown to be as low as 52 mK.^40^ Given that our excitation power is comparable, we estimate the temperature increase during our TERS measurements to be negligible (<1 K).

Overall, hyperspectral TERS mapping provided key insights into the VO_2_ thin film’s nanoscale structure, revealing local lattice deformations and polycrystalline regions with different crystallite orientations. These structural features are likely responsible for a higher density of grain boundaries in the VO_2_ thin films, which adversely affect their thermochromic performance—particularly in the context of smart window applications—by increasing the metal–insulator transition (MIT) temperature.

### 3.2 Nanoscale Structural Investigation of VO_2_/TiO_2_ Thin Film

We next performed nanoscale structural analysis of a VO_2_/TiO_2_ thin film deposited on a glass substrate using hyperspectral TERS imaging. VO_2_ thin films are commonly deposited on TiO_2_ layers to lower the MIT temperature of VO_2_ by inducing strain at the VO_2_/TiO_2_ interface.^41^ Remarkably, TERS imaging revealed the coexistence of two distinct TiO_2_ phases—anatase and brookite—within the thin film. Figure 4a–c depict TERS maps of the VO_2_/TiO_2_ thin film, showing the spatial distribution of the characteristic Raman signals corresponding to the VO_2_ M1 phase (222 cm^–1^), the TiO_2_ anatase phase (146 cm^–1^), and the TiO_2_ brookite phase (118 cm^–1^) with a step size of 50 nm. The composite map in Figure 4d provides a clear visualization of the spatial segregation of the VO_2_ M1, TiO_2_ anatase, and TiO_2_ brookite phases. Representative TERS spectra (averaged over 4 pixels) obtained from three distinct regions, marked in Figure 4d, are shown in Figure 4e. These spectra reveal characteristic peaks for anatase at 146 cm^–1^, 394 cm^–1^, 516 cm^–1^, and 636 cm^–1^, while the brookite phase exhibits distinct Raman modes at 118 cm^–1^, 141 cm^–1^, 197 cm^–1^, 261 cm^–1^, and 511 cm^–1^.^42–45^ The unique A_1g_ mode of the brookite phase at 118 cm^–1^ was critical for differentiating it from anatase phase. Detailed peak assignments for anatase and brookite phases are provided in Figure S5 and Table S2.

**Figure 4:**
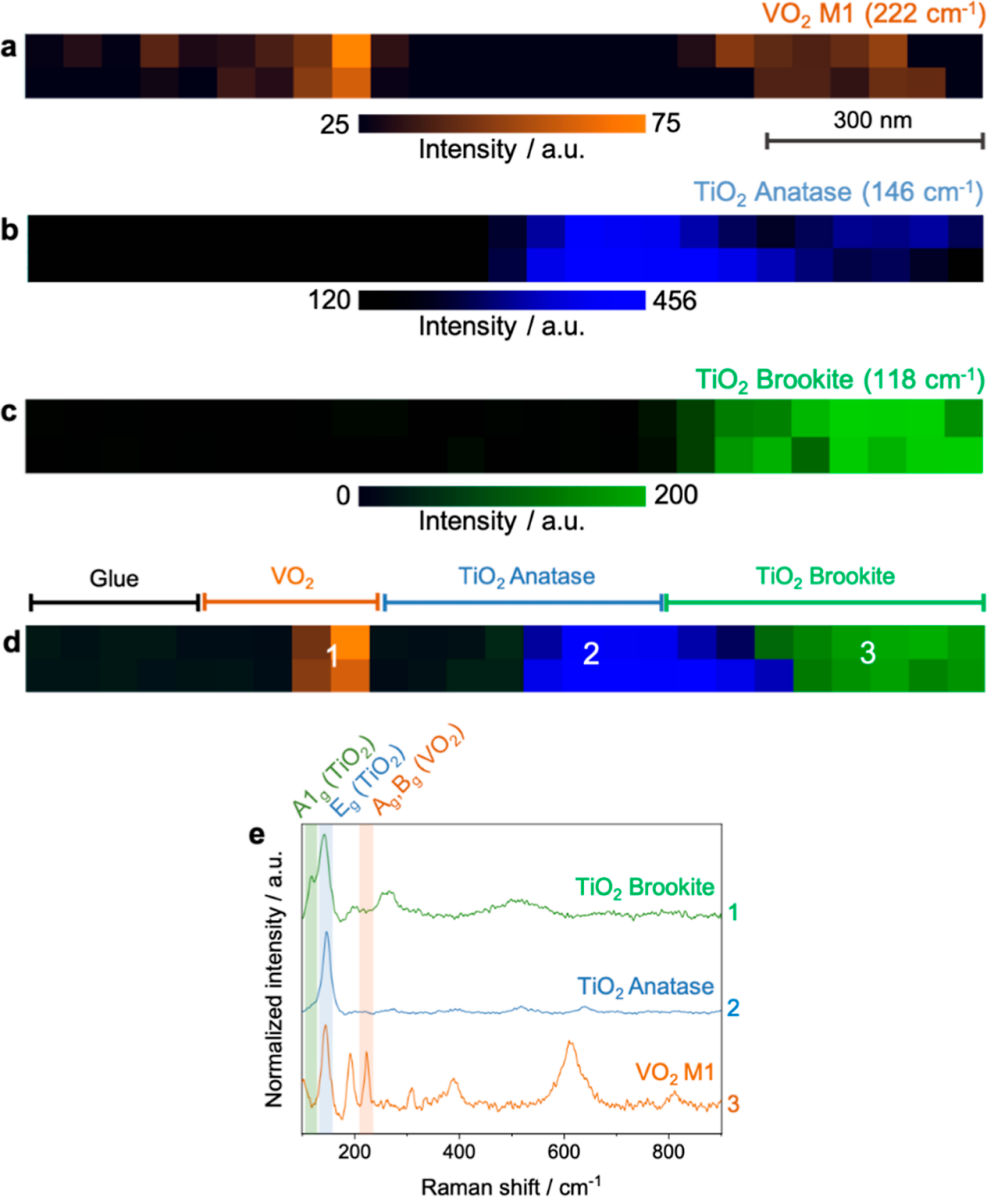
TERS maps illustrating the spatial distribution of Raman signals for (a) the VO_2_ M1 phase at 222 cm^–1^, (b) the TiO_2_ anatase phase at 146 cm^–1^, and (c) the TiO_2_ brookite phase at 118 cm^–1^, acquired with a step size of 50 nm. (d) Overlay of the TERS maps shown in panels a–c. (e) Representative TERS spectra (averaged over 4 pixels) collected from the locations labeled as 1–3 in panel d. It should be noted that the 146 cm^–1^ signal is common to both anatase and brookite phases, which results in an overestimation of the width of the anatase region in panel b.

While the anatase phase was expected due to the sample deposition parameters, the detection of the brookite phase was quite unexpected, given the difficulty of synthesizing pure brookite, which typically demands stringent control of precursor pH, deposition temperature, and substrate conditions.^46^ Furthermore, distinguishing anatase from brookite in mixed-phase TiO_2_ thin films has been reported to be particularly challenging due to overlapping Raman features.^47^ Nevertheless, the high sensitivity and nanoscale spatial resolution of TERS not only enabled the identification of the brookite phase but also allowed its spatial visualization within the TiO_2_ thin film. Furthermore, the A_1g_ Ti phonon mode at 141 cm^–1^ in the brookite phase exhibited greater broadening compared to the anatase phase, suggesting a lower degree of crystallinity in the brookite phase.^47,48^ Notably, no evidence of the TiO_2_ R phase was observed in our TERS measurements, presumably due to its low thermodynamic stability under the experimental conditions.^49^ Another significant observation was the red shift of the two A_1g_ Raman bands for brookite, located at 118 cm^–1^ and 141 cm^–1^, by 10 cm^–1^ and 12 cm^–1^, respectively, relative to previously reported values (Figure S5).^50^ This red shift is indicative of tensile strain in the brookite phase. However, it should be noted that quantitative interpretation of Raman shifts in terms of strain is rather challenging, particularly for the brookite phase of TiO_2_, where many material-specific parameters are not yet well established. Nevertheless, a shift of 10–12 cm^–1^ generally suggests the presence of moderate to strong local strain, especially in nanostructures or thin films where lattice mismatch and interfacial effects are significant. For comparison, previous studies on anatase-phase TiO_2_ have reported Raman shifts of up to 6 cm^–1^ in small crystallites,^51^ potentially attributable to strain. However, a precise quantitative correlation between Raman shift and strain requires further investigation.

These findings were consistently reproduced in TERS mapping of a different region, as shown in Figure S6. This map, acquired with a finer spatial step size of 10 nm, confirmed the coexistence of anatase and brookite phases within the TiO_2_ layer of the VO_2_/TiO_2_ thin film. Notably, the TERS spectra transitioned abruptly within a single 10 nm pixel from the anatase to the brookite phase, signifying a spatial resolution of 10 nm or better for the TERS measurements. Similar to the results presented in Figure 4, the brookite Raman peaks exhibited a red-shift, indicative of tensile strain, and the VO_2_/TiO_2_ interface was again observed to be diffuse, with evidence of interdiffusion between VO_2_ and TiO_2_ layers.

These observations suggest that the TiO_2_ layer predominantly consists of the anatase phase with high degree of crystallinity, interspersed with small quantities of brookite as an impurity. The tensile strain detected in the brookite phase appears to arise from interactions with the surrounding anatase matrix. Furthermore, the brookite phase is present throughout the TiO_2_ layer rather than being localized to the initial stages of film deposition as shown in the Figure S6. Interestingly, no evidence of additional VO_2_ phases or variations in crystal orientation was observed in the bilayer sample. The underlying reason for this absence is unclear.

We also conducted nanoscale investigation of the VO_2_/TiO_2_ interface using two-dimensional (2D) hyperspectral TERS imaging. Figure 5a–c present 2D TERS maps acquired in the interfacial region of the VO_2_/TiO_2_ thin film with a step size of 20 nm. Representative TERS spectra from locations marked as (i) and (ii) in Figure 5c are shown in Figure 5f, confirming the presence of the M1 phase in the VO_2_ region and the anatase phase in the TiO_2_ region. The corresponding TERS intensity profiles of the VO_2_ M1 and TiO_2_ anatase phases along the line indicated in Figure 5c are displayed in Figure 5g.

**Figure 5:**
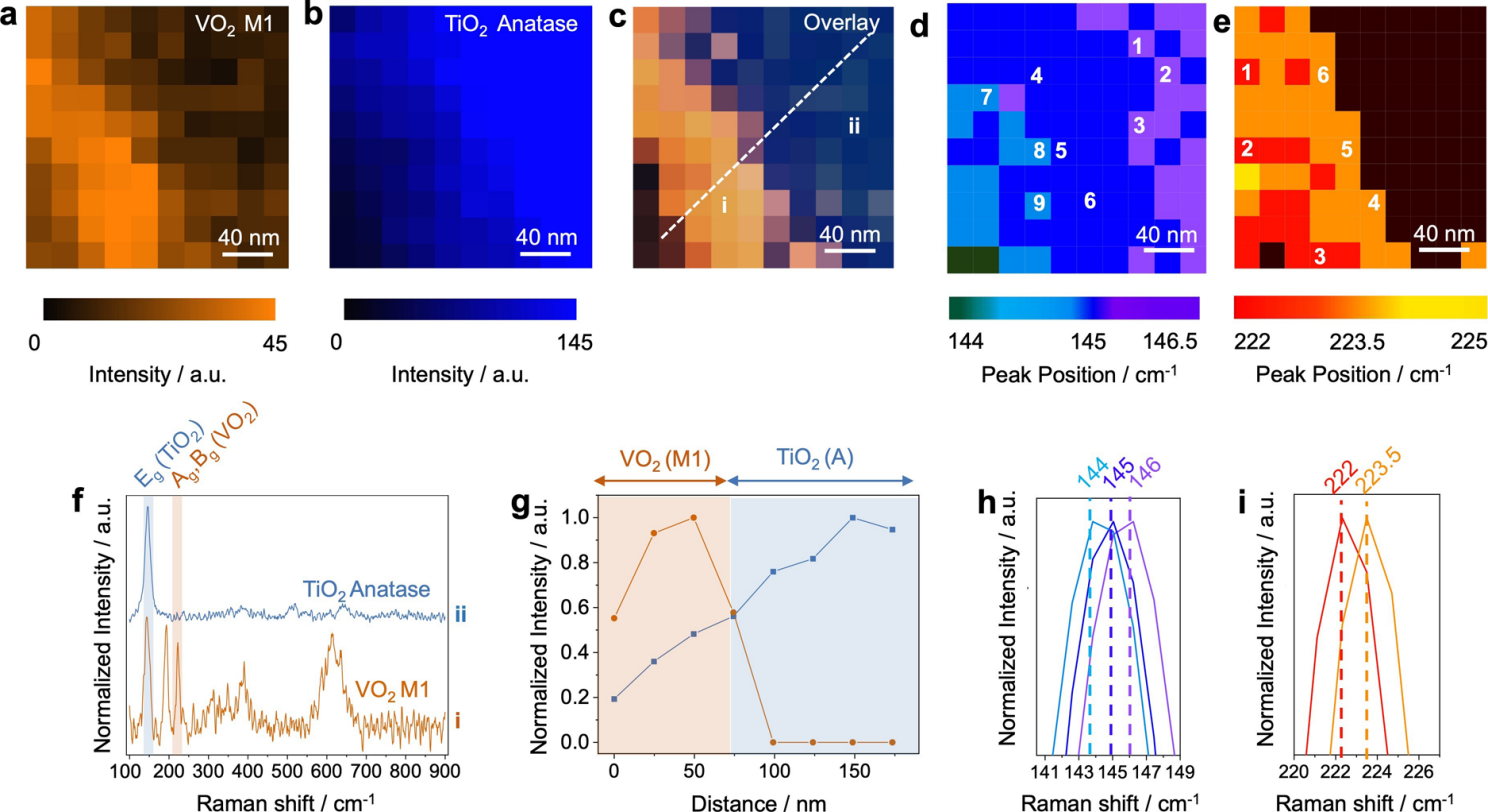
TERS maps of the VO_2_/TiO_2_ interface showing the intensity distribution of the Raman signals for (a) VO_2_ at 222 cm^–1^ and (b) TiO_2_ at 146 cm^–1^, acquired with a step size of 20 nm. (c) Overlay of the TERS maps from panels a and b, illustrating the spatial distribution of VO_2_ and TiO_2_ signals at the interface. TERS maps of the fitted peak positions of the (d) TiO_2_ Raman signal at ∼146 cm^–1^ and (e) VO_2_ Raman signal at ∼222 cm^–1^. All pixels with a S/N ratio below 5 were set to zero. (f) Representative TERS spectra collected from the VO_2_ and TiO_2_ regions at the locations labeled as i and ii in panel c confirming the presence of the M1 phase in the VO_2_ region and the anatase phase in the TiO_2_ region. (g) Intensity profiles of the near-field Raman signals corresponding to the VO_2_ M1 phase (orange) and the TiO_2_ anatase phase (blue) along the designated line in panel c. (h) Comparison of peak positions at the pixels labeled in panel d. Purple, blue and light blue spectra represent the average of pixels labeled as 1–3, 4–6, and 7–9, respectively. (i) Comparison of peak positions at the pixels labeled in panel e. Red and orange spectra represent the average of pixels labeled as 1–3, and 4–6, respectively.

Notably, the TERS intensity profile reveals a spatially extended Raman signal at the VO_2_/TiO_2_ interface, indicative of substantial interdiffusion between the two materials. As shown in Figure 5g (where pixels with a S/N ratio ≤5 are set to zero and marked with a star), the interdiffusion appears predominantly unidirectional—from TiO_2_ into VO_2_. This asymmetry likely originates from differences in oxygen partial pressure, defect chemistry, and the deposition sequence during PLD. TiO_2_ was deposited at a higher oxygen partial pressure (10 Pa), resulting in a stoichiometric, well-crystallized film with minimal oxygen vacancies.^52^ In contrast, VO_2_ was grown under a lower oxygen pressure (1 Pa), leading to a higher concentration of oxygen vacancies, which are known to enhance cation mobility by establishing chemical potential gradients.^53^ These gradients likely drive Ti ion migration into the VO_2_ layer during high-temperature growth.^54^ In addition to these thermodynamic considerations, the asymmetric signal distribution may be further influenced by the higher Raman scattering cross-section of TiO_2_ compared to VO_2_. This can result in enhanced far-field signal contributions from TiO_2_, thereby increasing its apparent spectral presence near the interface in TERS maps. While VO_2_ signals are also observed within the TiO_2_ region, their spatial extent is significantly more confined. In Figure 5g, TiO_2_ intermixing into VO_2_ is detected over a distance of up to ∼50 nm. However, this value should be interpreted as a qualitative estimate rather than a definitive measurement, as far-field contributions cannot be fully excluded, making a precise quantification of Ti interdiffusion challenging. A similar extent of intermixing was consistently observed in another region of the thin film, as shown in Figure S7.

A detailed analysis of the hyperspectral TERS data set was carried out to investigate the presence of strain in the VO_2_/TiO_2_ interfacial region. For this purpose, the two characteristic Raman peaks—146 cm^–1^ for TiO_2_ and 222 cm^–1^ for VO_2_—were fitted using a Gaussian–Lorentzian function. Only spectra with a S/N ratio ≥5 were included in the peak fitting analysis to ensure data reliability. Spatially resolved TERS maps of the fitted peak positions are presented in Figure 5d,e. Notably, the TiO_2_ Raman peak exhibited a red shift from the bulk toward the VO_2_/TiO_2_ interface, as illustrated in Figure 5d,h. This systematic shift indicates an increase in tensile strain within the TiO_2_ crystallites near the interface. In contrast, the VO_2_ Raman peak displayed a blue shift within the mapped region. However, unlike TiO_2_, the VO_2_ peak shifts did not exhibit a consistent spatial trend, as shown in Figure 5e,i. Similar nanoscale strain effects were observed at other interfacial locations (Figure S7), where TiO_2_ consistently displayed a red shift from the bulk toward the interface, while the VO_2_ peak shifts remained spatially irregular. These findings reveal distinct strain dynamics at the VO_2_/TiO_2_ interface, where TiO_2_ undergoes a well-defined tensile strain gradient, while VO_2_ exhibits localized and heterogeneous strain variations, likely concentrated at the grain boundaries of its nanocrystallites.^27,28^

Furthermore, these observations indicate that the PLD process used for the growth of VO_2_ on TiO_2_ yields a polycrystalline film, resulting in significant localized nanoscale structural heterogeneity. Such nanoscale insights into intermixing and strain could not be achieved using conventional vibrational spectroscopy, which is limited by low sensitivity and diffraction-limited spatial resolution. These findings underscore the power of TERS in unraveling localized structural and interfacial properties at the nanoscale.

## 4 Conclusions

In summary, this study presents nanoscale structural analysis of VO_2_ and VO_2_/TiO_2_ thin films using hyperspectral TERS imaging, revealing novel insights into their phase composition, crystallite orientation, strain distribution, and interfacial characteristics. In pristine VO_2_ thin films, TERS mapping uncovered a homogeneously distributed M1 phase and localized lattice deformations and regions of nanocrystallites with different orientations, resulting in a high density of grain boundaries. These structural features are likely to increase the MIT temperature of these VO_2_ thin films, adversely impacting their thermochromic performance in smart window applications.

In the VO_2_/TiO_2_ thin-film system, TERS imaging revealed the coexistence of anatase and brookite phases within the TiO_2_ layer, with the brookite phase exhibiting tensile strain and lower crystallinity compared to anatase. The nanoscale spatial resolution of TERS enabled the precise identification and mapping of these phases, a task challenging for conventional analytical tools. Furthermore, the VO_2_/TiO_2_ interface was found to be rough, exhibiting nanoscale intermixing between the VO_2_ and TiO_2_ layers, along with strain-induced shifts in their Raman signals. Notably, TiO_2_ exhibited a well-defined tensile strain gradient, while VO_2_ displayed localized and spatially heterogeneous strain variations within the VO_2_/TiO_2_ interfacial region.

By providing novel nanoscale insights into the structural and interfacial properties of VO_2_ and VO_2_/TiO_2_ thin films, this work demonstrates the potential of TERS in advancing the understanding and optimization of thermochromic materials for energy-efficient applications. These findings have important implications for the design of next-generation thermochromic smart coatings and devices, where precise control of nanoscale structure and strain is critical for achieving optimal functionality.

## Supplementary Material



## Data Availability

The original data used in this publication are made available in a curated data archive at ETH Zurich (https://www.researchcollection.ethz.ch) under the DOI: 10.3929/ethz-b-000713806.

## Notes

TERS and far-field comparison, representative VO_2_ spectra, tentative assignment of the Raman signal VO_2_ thin film, far-field measurements as a function of laser power, far-field measurements as a function of an acquisition time, representative TiO_2_ spectra (brookite and anatase), additional two TERS maps from the interfacial region (PDF)

## References

[1] Mitchell J. F., Lowe J., Wood R. A., Vellinga M. (2006). Extreme events due
to human-induced climate change. Philos. Trans.
R. Soc., A.

[2] Santamouris M., Papanikolaou N., Livada I., Koronakis I., Georgakis C., Argiriou A., Assimakopoulos D. N. (2001). On the
impact of urban climate on the energy consumption of buildings. J. Sol. Energy.

[3] Kolokotroni M., Zhang Y., Watkins R. (2007). The London Heat Island
and building
cooling design. J. Sol. Energy.

[4] Morin F. J. (1959). Oxides
Which Show a Metal-to-Insulator Transition at the Neel Temperature. Phys. Rev. Lett..

[5] Greenberg C. B. (1994). Optically
switchable thin films: a review. Thin Solid
Films.

[6] MacChesney J. B., Potter J. F., Guggenheim H. J. (1968). Preparation
and Properties of Vanadium
Dioxide Films. J. Electrochem. Soc..

[7] Mitsuishi T. (1967). On the Phase
Transformation of VO2. Jpn. J. Appl. Phys..

[8] Bernhoft R. A. (2012). Mercury
Toxicity and Treatment: A Review of the Literature. J. Environ. Public Health.

[9] Kumar S., Qadir A., Maury F., Bahlawane N. (2017). Visible Thermochromism
in Vanadium Pentoxide Coatings. ACS Appl. Mater.
Interfaces.

[10] Roppolo I., Celasco E., Fargues A., Garcia A., Revaux A., Dantelle G., Maroun F., Gacoin T., Boilot J.-P., Sangermano M. (2011). Luminescence thermochromism of acrylic materials
incorporating copper iodide clusters. J. Mater.
Chem..

[11] Wang X., Narayan S. (2021). Thermochromic Materials
for Smart Windows: A State-of-Art
Review. Front. Energy Res..

[12] Hakami A., Srinivasan S. S., Biswas P. K., Krishnegowda A., Wallen S. L., Stefanakos E. K. (2022). Review on thermochromic materials:
development, characterization, and applications. J. Coat. Technol. Res..

[13] Bayati M. R., Molaei R., Wu F., Budai J. D., Liu Y., Narayan R. J., Narayan J. (2013). Correlation between structure and
semiconductor-to-metal transition characteristics of VO2/TiO2/sapphire
thin film heterostructures. Acta Mater..

[14] Lappalainen J., Heinilehto S., Saukko S., Lantto V., Jantunen H. (2008). Microstructure
dependent switching properties of VO2 thin films. Sens. Actuators, A.

[15] Mayer J., Giannuzzi L. A., Kamino T., Michael J. (2007). TEM Sample
Preparation
and FIB-Induced Damage. MRS Bull..

[16] Shen, Y. Q. ; Li, L. H. ; Tee, I. ; Lee, K. W. ; Chen, Y. ; Zhu, J. ; Zhao, S. P. A study of coating techniques for ultra-thin film X-TEM sample preparation. In IEEE 24th International Symposium on the Physical and Failure Analysis of Integrated Circuits (IPFA), 2017; pp 1–4.

[17] Bienz S., Spaggiari G., Calestani D., Trevisi G., Bersani D., Zenobi R., Kumar N. (2024). Nanoscale Chemical Analysis of Thin
Film Solar Cell Interfaces Using Tip-Enhanced Raman Spectroscopy. ACS Appl. Mater. Interfaces.

[18] Stöckle R. M., Suh Y. D., Deckert V., Zenobi R. (2000). Nanoscale
chemical
analysis by tip-enhanced Raman spectroscopy. Chem. Phys. Lett..

[19] Hartschuh A. (2008). Tip-Enhanced
Near-Field Optical Microscopy. Angew. Chem.,
Int. Ed..

[20] Mayer K. M., Hafner J. H. (2011). Localized Surface Plasmon Resonance Sensors. Chem. Rev..

[21] Dzhagan V. M., Valakh M. Y., Isaieva O. F., Yukhymchuk V. O., Stadnik O. A., Gudymenko O. Y., Lytvyn P. M., Kulbachynskyi O. A., Yefanov V. S., Romanyuk B. M. (2024). Raman fingerprints of
different vanadium oxides as impurity phases in VO2 films. Opt. Mater..

[22] Schilbe P. (2002). Raman scattering
in VO2. Phys. B.

[23] Wu H., Fu Q., Bao X. (2016). In situ Raman spectroscopy study
of metal-enhanced
hydrogenation and dehydrogenation of VO2. J.
Condens. Matter Phys..

[24] Becker M., Kuhl F., Hauptmann J., Kessler J., Benz S. L., Chatterjee S., Polity A., Klar P. J. (2023). Employing Ion-Beam
Sputter Deposited TiO2 Buffer Layers for VO2-Related Devices. ACS Appl. Electron. Mater..

[25] Chang T., Zhu Y., Huang J., Luo H., Jin P., Cao X. (2021). Flexible VO2
thermochromic films with narrow hysteresis loops. Sol. Energy Mater. Sol. Cells.

[26] Shepelin N. A., Tehrani Z. P., Ohannessian N., Schneider C. W., Pergolesi D., Lippert T. (2023). A practical guide to
pulsed laser
deposition. Chem. Soc. Rev..

[27] Rai, A. ; Istrate, C. ; Socol, G. ; Iacob, N. ; Hansen, V. ; Mihailescu, C. N. ; Kuncser, V. ; Delimitis, A. Correlation of Nanostructural Features with Phase Transition in Thermochromic VO2 Thin Films for Smart Windows Applications. In Analytical and Experimental Methods in Mechanical and Civil Engineering; Springer Nature Switzerland, 2024; pp 3–12.

[28] Rai A., Iacob N., Leca A., Locovei C., Kuncser V., Mihailescu C. N., Delimitis A. (2022). Microstructural Investigations of
VO2 Thermochromic Thin Films Grown by Pulsed Laser Deposition for
Smart Windows Applications. Inorganics.

[29] Shvets P., Dikaya O., Maksimova K., Goikhman A. (2019). A review of Raman spectroscopy
of vanadium oxides. J. Raman Spectrosc..

[30] Zhang C., Yang Q., Koughia C., Ye F., Sanayei M., Wen S.-J., Kasap S. (2016). Characterization of
vanadium oxide
thin films with different stoichiometry using Raman spectroscopy. Thin Solid Films.

[31] Vilanova-Martínez P., Hernández-Velasco J., Landa-Cánovas A. R., Agulló-Rueda F. (2016). Laser heating
induced phase changes of VO2 crystals
in air monitored by Raman spectroscopy. J. Alloys
Compd..

[32] Olivia
Avilés M., Wang Z., Sham T.-K., Lagugné-Labarthet F. (2023). On the oxidation
of VS2 2D platelets using tip-enhanced Raman spectroscopy. Curr. Opin. Solid State Mater. Sci..

[33] Marini C., Arcangeletti E., Di Castro D., Baldassare L., Perucchi A., Lupi S., Malavasi L., Boeri L., Pomjakushina E., Conder K. (2008). Optical
properties of
V_1‑x_Cr_x_O_2_ compounds under
high pressure. Phys. Rev. B: Condens. Matter
Mater. Phys..

[34] Strelcov E., Tselev A., Ivanov I., Budai J. D., Zhang J., Tischler J. Z., Kravchenko I., Kalinin S. V., Kolmakov A. (2012). Doping-Based
Stabilization of the M2 Phase in Free-Standing VO2 Nanostructures
at Room Temperature. Nano Lett..

[35] Bleu Y., Bourquard F., Misdanitis K., Poulet A., Loir A.-S., Garrelie F., Donnet C. (2023). Polymorphism of VO2 thin film: M1,
T, and M2 single phase synthesis using pulsed laser deposition. Mater. Today Commun..

[36] Strelchuk V. V., Kolomys O. F., Maziar D. M., Melnik V. P., Romanyuk B. M., Gudymenko O. Y., Dubikovskyi O. V., Liubchenko O. I. (2024). Effect
of structural disorder on the modification of V–V and V–O
bond lengths at the metal-dielectric phase transition in VO2 thin
films. Mater. Sci. Semicond. Process..

[37] Basu R., Patsha A., Chandra S., Amirthapandian S., Karkala Gururaj R., Dasgupta A., Dhara S. (2019). Polarized Tip-Enhanced
Raman Spectroscopy in Understanding Metal-to-Insulator and Structural
Phase Transition in VO2. J. Phys. Chem. C.

[38] Mussi V., Bovino F. A., Falsini R., Daloiso D., Lupo F. V., Kunjumon R., Voti R. L., Cesca T., Macaluso R., Sibilia C. (2024). Polarized
Raman mapping and phase-transition by CW
excitation for fast purely optical characterization of VO2 thin films. Sci. Rep..

[39] Huang F., Gong M., Tian S., Zhao X., Liu B. (2022). Controlling
the crystalline orientation and textual morphologies of the VO2 film
and the effect on insulator–metal transition properties. Jpn. J. Appl. Phys..

[40] Wang R., Li J., Rigor J., Large N., El-Khoury P. Z., Rogachev A. Y., Kurouski D. (2020). Direct Experimental Evidence of Hot
Carrier-Driven Chemical Processes in Tip-Enhanced Raman Spectroscopy
(TERS). J. Phys. Chem. C.

[41] Lu H., Li L., Tang Z., Xu M., Zheng Y., Becker M., Lu Y., Li M., Li P., Zhang Z. (2023). Correlation
of metal-to-insulator transition and strain state of VO2 thin films
on TiO2 (110) substrates. Appl. Phys. Lett..

[42] Scepanovic J., Grujić-Brojčin M., Dohcevic-Mitrovic Z., Popović Z. V. (2009). Characterization of anatase TiO2 nanopowder by variable-temperature
Raman spectroscopy. Sci. Sinter..

[43] Zhang W. F., He Y. L., Zhang M. S., Yin Z., Chen Q. (2000). Raman scattering
study on anatase TiO2 nanocrystals. J. Phys.
D: Appl. Phys..

[44] Ohsaka T., Izumi F., Fujiki Y. (1978). Raman spectrum
of anatase, TiO2. J. Raman Spectrosc..

[45] Tompsett G. A., Bowmaker G. A., Cooney R. P., Metson J. B., Rodgers K. A., Seakins J. M. (1995). The Raman spectrum
of brookite, TiO2 (Pbca, Z = 8). J. Raman Spectrosc..

[46] Gonullu M. P., Ates H. (2020). Investigation of the
impact of annealing on the structural, optical
and morphological evolution of mixture-phase ALD-TiO2 films containing
brookite. Superlattice. Microst..

[47] Ceballos-Chuc M. C., Ramos-Castillo C. M., Alvarado-Gil J. J., Oskam G., Rodríguez-Gattorno G. (2018). Influence
of Brookite Impurities on the Raman Spectrum of TiO2 Anatase Nanocrystals. J. Phys. Chem. C.

[48] Kremenović A., Grujić-Brojčin M., Tomić N., Lazović V., Bajuk-Bogdanović D., Krstić J., Šćepanović M. (2022). Size-strain
line-broadening analysis
of anatase/brookite (TiO(2))-based nanocomposites with carbon (C):
XRPD and Raman spectroscopic analysis. Acta
Crystallogr., Sect. B: Struct. Sci., Cryst. Eng. Mater..

[49] Sharma A. K., Thareja R. K., Willer U., Schade W. (2003). Phase transformation
in room temperature pulsed laser deposited TiO2 thin films. Appl. Surf. Sci..

[50] Li J.-G., Ishigaki T. (2004). Brookite→rutile phase transformation
of TiO2
studied with monodispersed particles. Acta Mater..

[51] Alhomoudi I. A., Newaz G. (2009). Residual stresses and
Raman shift relation in anatase TiO2 thin film. Thin Solid Films.

[52] Park Y., Sim H., Jo M., Kim G. Y., Yoon D., Han H., Kim Y., Song K., Lee D., Choi S. Y. (2020). Directional
ionic transport across the oxide interface enables low-temperature
epitaxy of rutile TiO(2). Nat. Commun..

[53] Sim H., Doh K.-Y., Park Y., Song K., Kim G.-Y., Son J., Lee D., Choi S.-Y. (2024). Crystallographic Pathways to Tailoring
Metal-Insulator Transition through Oxygen Transport in VO2. Small.

[54] Maekawa K., Takizawa M., Wadati H., Yoshida T., Fujimori A., Kumigashira H., Oshima M., Muraoka Y., Nagao Y., Hiroi Z. (2007). Photoemission
study of TiO2/VO2 interfaces. Phys. Rev. B:
Condens. Matter Mater. Phys..

