# Supplementary material for: Nanoscale Structural Insights into Thermochromic VO2 Thin Films Using Tip-Enhanced Raman Spectroscopy
Source: ACS Appl Mater Interfaces. 2025 May 22;17(22):32625–34. doi: 10.1021/acsami.5c04827 (PMC12147077; doi:10.1021/acsami.5c04827)
Supplement: Supplementary file 1 [file am5c04827_si_001.pdf]

Supporting Information

Nanoscale Structural Insights into Thermochromic VO_2 Thin-Films using Tip-Enhanced Raman Spectroscopy

Ayushi Rai[a§], Siiri Bienz[b§], Vidar F. Hansen[a], Renato Zenobi[b*], and Naresh Kumar[b*]

[(a)]Department of Mechanical and Structural Engineering and Materials Science, University of Stavanger, N-4036 Stavanger, Norway

[(b)]Department of Chemistry and Applied Biosciences, ETH Zurich, Vladimir-Prelog-Weg 1–5/10, 8093 Zurich, Switzerland

*Email: zenobi@org.chem.ethz.ch, naresh.kumar@org.chem.ethz.ch

Figure S1 Comparison of TERS and far-field Raman signals.

Figure S2 Representative TERS spectra of VO_2 thin film.

Table S1 Tentative assignment of bands in the Raman spectrum of VO_2 thin film.

Figure S3 Far-field Raman measurements of VO_2 thin film as a function of laser power.

Figure S4 Far-field measurements of VO_2 thin film as a function of acquisition time.

Figure S5 Representative TERS spectra of TiO_2 Brookite and Anatase thin film.

Table S2 Tentative assignment of bands in the Raman spectrum of TiO_2 Brookite and Anatase phases.

Figure S6 Additional TERS map recorded at the VO_2/TiO_2 interfacial region

Figure S7 Additional TERS map of the VO_2/TiO_2 interface region.

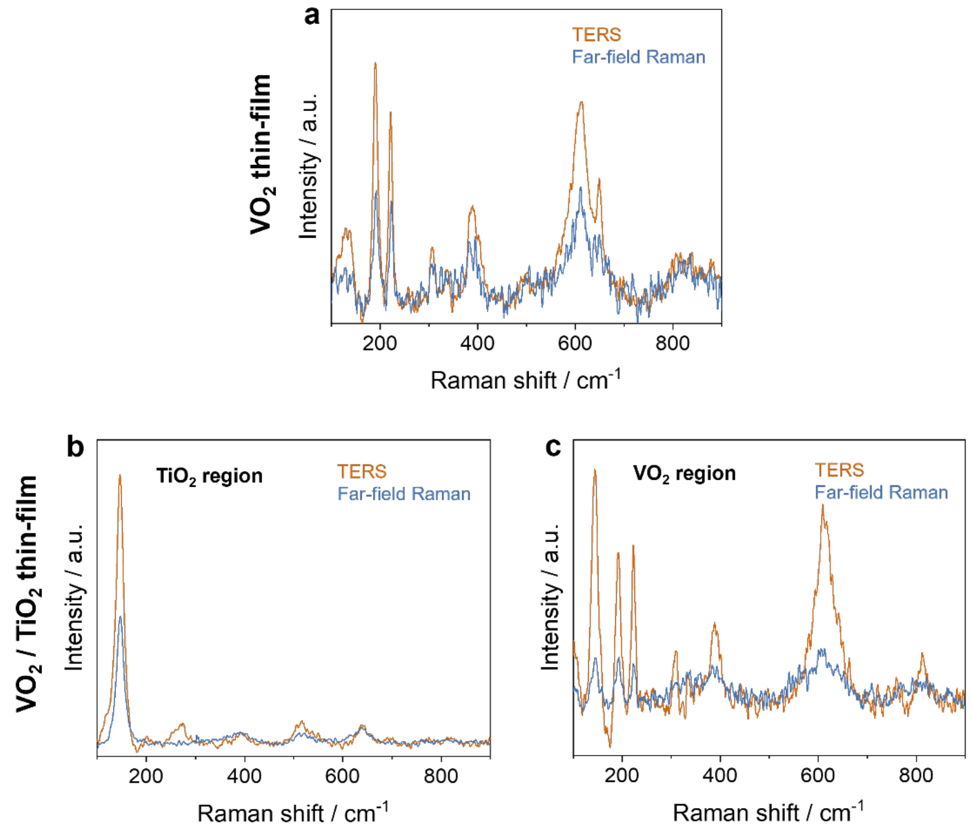

Figure S1. (a) Comparison of the TERS (orange) and far-field Raman (blue) spectra acquired at the same location on VO_2 thin-film on glass substrate. Comparison of the TERS and far-field Raman spectra measured in (b) the TiO_2 region and (c) the VO_2 region of VO_2/TiO_2 thin-film on glass substrate. The VO_2 and VO_2/TiO_2 thin-film samples were analyzed using different TERS probes, which displayed different signal enhancements. For all measurements, the TERS and far-field Raman spectra were obtained under identical conditions, including acquisition time and laser power. All spectra were smoothened and background-subtracted to facilitate comparison. The enhanced Raman signals in the TERS spectra confirm the plasmonic sensitivity of the Ag-coated TERS probes.

In TERS measurements, "Contrast" is a key parameter to assess the plasmonic enhancement of Raman signals. It is defined as $(I_{TERS} - I_{Far\text{-}field})/ I_{Far\text{-}field}$, where I_{TERS} represents the intensity of the TERS signal, and $I_{Far\text{-}field}$ corresponds to the intensity of the far-field Raman signal.[1] In the measurements presented in Panels a–c, a Contrast of up to 4.3 was achieved, highlighting the strong plasmonic sensitivity of our Ag-coated TERS probes.

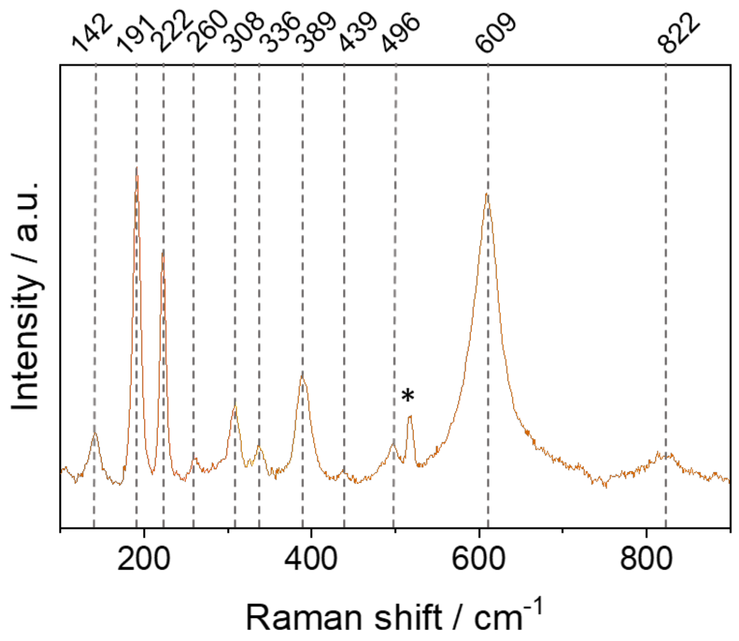

Figure S2. Representative TERS spectrum of VO_2 thin-film on glass substrate. The major VO_2 Raman peaks are annotated with their corresponding vibrational modes, and the characteristic Silicon (Si) peak is denoted by an asterisk (*). Detailed peak assignments are provided in Table S1.

Table S1. Tentative assignment of Raman peaks in the TERS spectra of VO_2 thin-film.[2]

**Peak position (cm^{-1})**	**Vibrational mode**	**Compound and phase**
142	B_g	VO_2 (M1)
191	A_g	VO_2 (M1)
222	A_g, B_g	VO_2 (M1)
260	B_g	VO_2(M1)
308	A_g	VO_2(M1)
336	A_g	VO_2(M1)
389	A_g, B_g	VO_2(M1)
439	B_g	VO_2(M1)
496	A_g	VO_2(M1)
517		Si
609	A_g	VO_2 (M1)
822	B_g	VO_2 (M1)

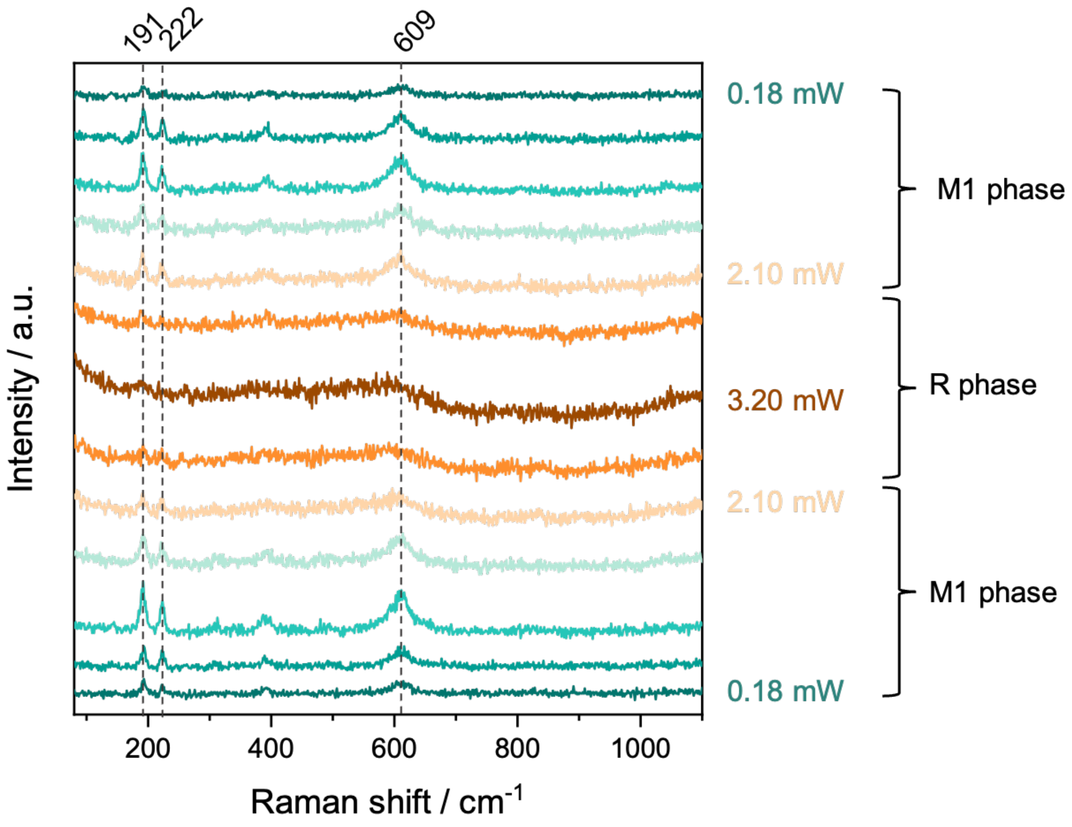

Figure S3. Far-field Raman spectra of the VO_2 thin-film acquired under varying 532 nm excitation laser powers. The phase transition from the M1 phase to the R phase (MIT) initiates at approximately 2.1 mW and is complete by around 3.2 mW. The phase transition was fully reversible upon reducing the laser power. Importantly, no Raman peaks associated with the oxidized phases VO_2 were detected during the transition process.

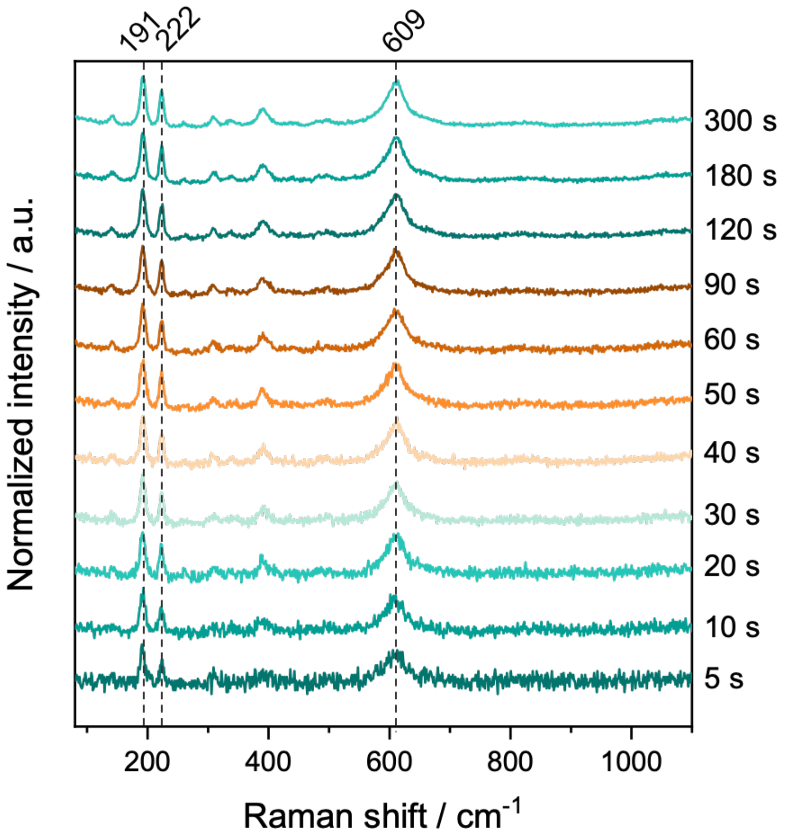

Figure S4. Far-field Raman spectra of the VO_2 thin-film recorded at different integration times. Laser power: 263 µW. Notably, no Raman peaks indicative of VO_2 oxidation or spectral changes associated with the M1 → R phase transition was observed as the acquisition time increased.

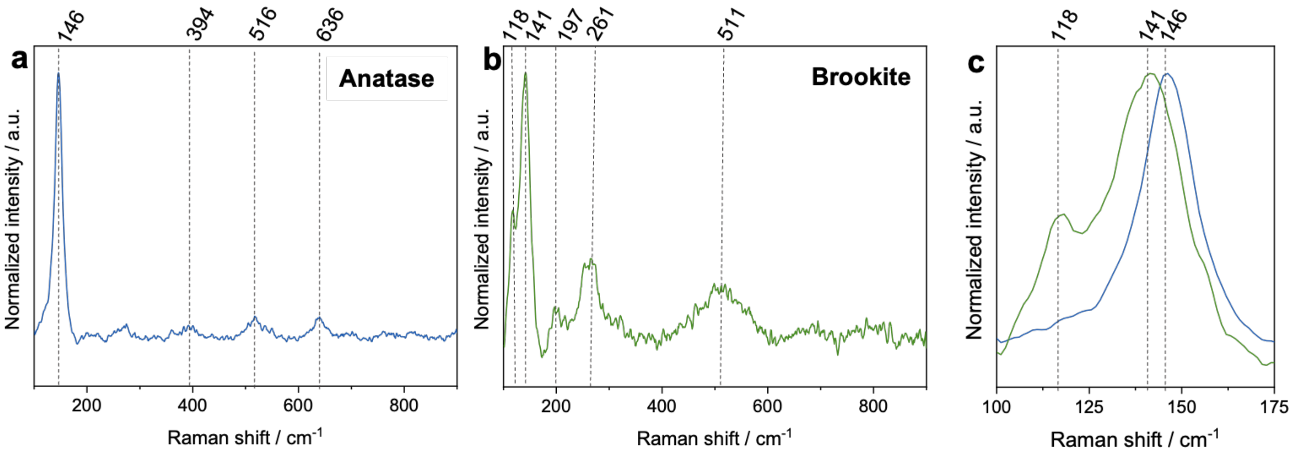

Figure S5. Representative TERS spectra acquired from regions of the VO_2/TiO_2 thin film exhibiting the TiO_2 (a) anatase and (b) brookite phases. (c) Comparison of the TERS spectra corresponding to the TiO_2 anatase and brookite phases. The brookite phase is characterized by two red-shifted A_{1g} modes at 118 cm^{-1} and 141 cm^{-1}, which serve as key identifiers to distinguish it from the anatase phase. Detailed Raman peak assignments are provided in Table S2.

Table S2. Tentative assignment of Raman peaks in the TERS spectra of TiO_2 thin-film.[3,4,5,6,7]

Raman peak (cm^{-1})	Vibrations	Phase
118	A_{1g}	TiO_2 (B)
141	A_{1g}	TiO_2 (B)
146	E_g	TiO_2 (A)
197	A_{1g}	TiO_2 (B)
261	B_{3g}	TiO_2 (B)
394	B_{1g}	TiO_2 (A)
511	B_{3g}	TiO_2 (B)
516	A_{1g}/B_{1g}	TiO_2 (A)
636	E_g	TiO_2 (A)

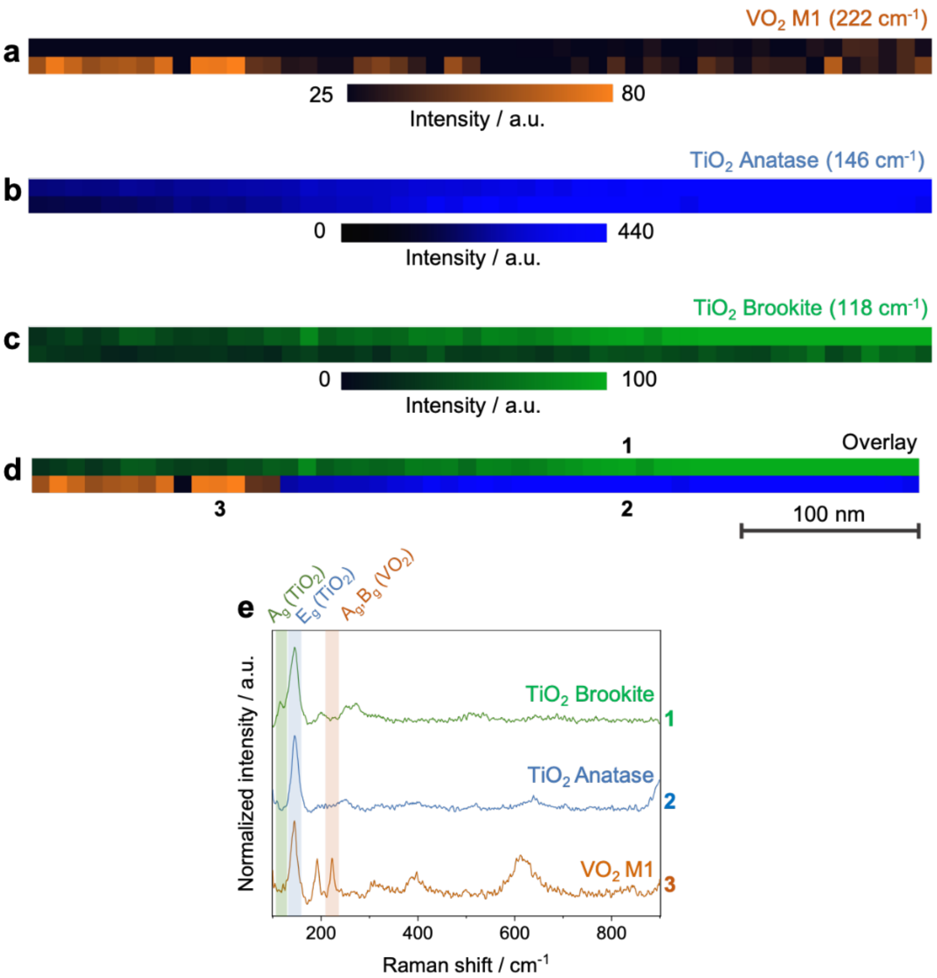

Figure S6. TERS maps depicting the spatial intensity distribution of (a) the VO_2 M1 phase Raman signal at 222 cm^{-1}, (b) the TiO_2 anatase phase Raman signal at 146 cm^{-1}, and (c) the TiO_2 brookite phase Raman signal at 118 cm^{-1}, acquired with a step size of 10 nm. (d) Composite overlay of the TERS maps from Panels a–c, highlighting the spatial separation of the respective phases. (e) Representative TERS spectra collected from the locations labelled as 1-3 Panel d. Additionally, the A_{1g} and E_g Raman modes of the TiO_2 brookite phase at 118 cm^{-1} and 146 cm^{-1} exhibited a red-shift of approximately 9 cm^{-1} compared to previously reported values, indicative of tensile strain in this phase.[7,8] Conversely, the TiO_2 anatase phase displayed no detectable peak shifts, signifying the absence of strain in its structure.

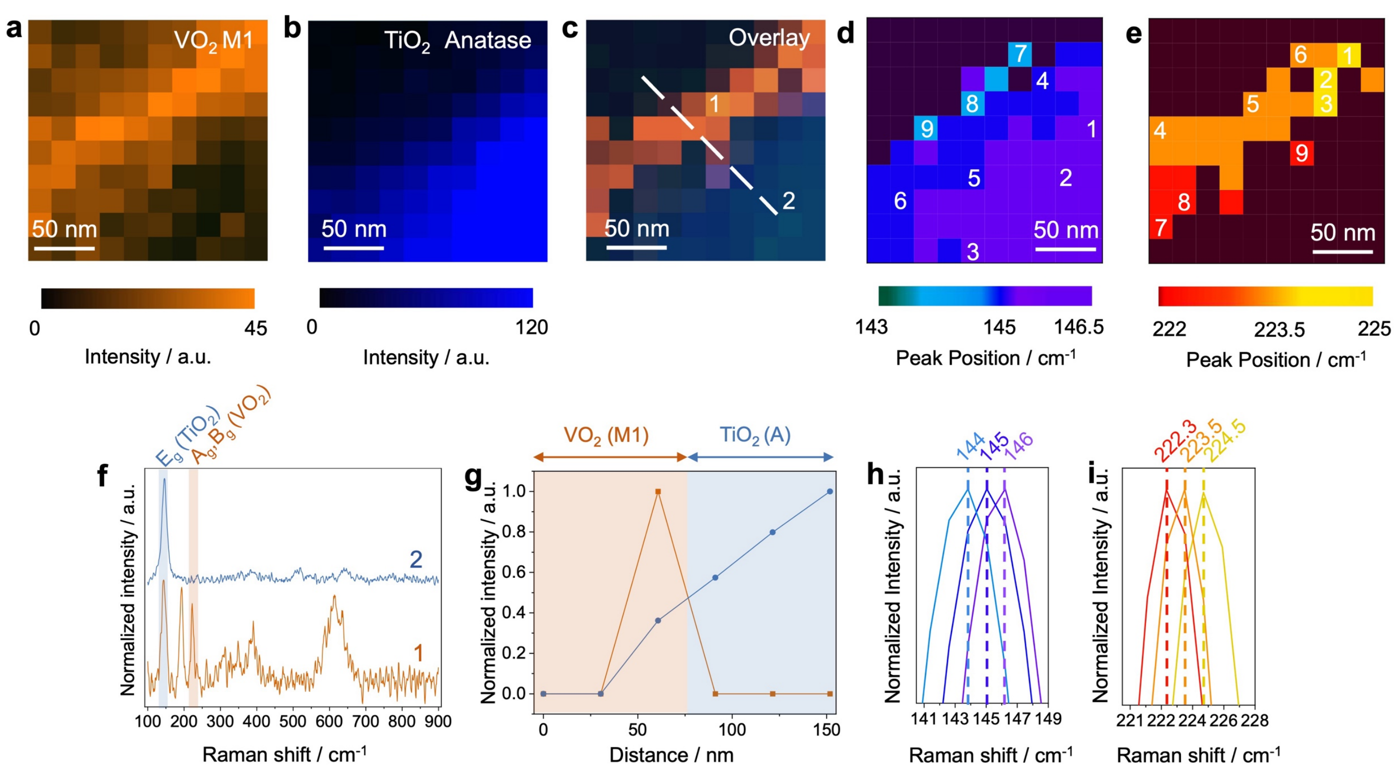

Figure S7. 2D TERS maps of another VO_2/TiO_2 interfacial region, highlighting the intensity distribution of (a) the VO_2 Raman signal at 222 cm^{-1} and (b) the TiO_2 Raman signal at 146 cm^{-1}. Both maps were acquired with a step size of 20 nm. (c) Overlay of the VO_2 and TiO_2 TERS maps presented in Panels a and b illustrating their spatial distribution at the interface. TERS maps of the fitted peak positions of the (d) TiO_2 Raman signal at ~146 cm^{-1} and (e) VO_2 Raman signal at ~222 cm^{-1}. All pixels with a signal-to-noise ratio below 5 were set to zero (dark brown) at (d) 146 cm^{-1} and (e) 222 cm^{-1}. (f) Representative TERS spectra collected from locations 1 and 2 marked in panel (c), confirming the presence of the VO_2 M1 phase and the TiO_2 anatase phase, respectively. (g) Intensity profiles of the near-field Raman signals corresponding to VO_2 M1 phase (orange) and the TiO_2 anatase phase (blue) along the white line in panel (c), revealing nanoscale intermixing of compounds at the VO_2/TiO_2 interface. (h) Comparison of the peak positions obtained from the average of three pixels in the corresponding regions labeled in Panel d for the fitted peak of the TiO_2 and (i) VO_2 main Raman modes. The TiO_2 peak is averaged over pixels 1–3 (purple), 4–6 (blue), and 7–9 (light blue/green), while the VO_2 peak is averaged over pixels 1–3 (yellow), 4–6 (red), and 7–9 (orange), highlighting spectral variations across the interfacial region.

Supporting references

1) Stadler, J.; Schmid, T.; Zenobi, R. Developments in and practical guidelines for tip-enhanced Raman spectroscopy. *Nanoscale* **2012**, *4* (6), 1856-1870.
2) Shvets, P.; Dikaya, O.; Maksimova, K.; Goikhman, A. A review of Raman spectroscopy of vanadium oxides. *J. Raman Spectrosc.* **2019**, *50* (8), 1226-1244.
3) Tompsett, G. A.; Bowmaker, G. A.; Cooney, R. P.; Metson, J. B.; Rodgers, K. A.; Seakins, J. M. The Raman spectrum of brookite, TiO2 (Pbca, Z = 8). *J. Raman Spectrosc.* **1995**, *26* (1), 57-62.
4) Scepanovic, J.; Grujić-Brojčin, M.; Dohcevic-Mitrovic, Z.; Popović, Z. V. Characterization of anatase TiO2 nanopowder by variable-temperature Raman spectroscopy. *Sci. Sinter.* **2009**, *41*, 67-73.
5) Zhang, W. F.; He, Y. L.; Zhang, M. S.; Yin, Z.; Chen, Q. Raman scattering study on anatase TiO2 nanocrystals. *J. Phys. D: Appl. Phys.* **2000**, *33* (8), 912.
6) Ohsaka, T.; Izumi, F.; Fujiki, Y. Raman spectrum of anatase, TiO2. *J. Raman Spectrosc.* **1978**, *7* (6), 321-324.
7) Ceballos-Chuc, M. C.; Ramos-Castillo, C. M.; Alvarado-Gil, J. J.; Oskam, G.; Rodríguez-Gattorno, G. Influence of Brookite Impurities on the Raman Spectrum of TiO2 Anatase Nanocrystals. *J. Phys. Chem. C* **2018**, *122* (34), 19921-19930.
8) Kremenović, A.; Grujić-Brojčin, M.; Tomić, N.; Lazović, V.; Bajuk-Bogdanović, D.; Krstić, J.; Šćepanović, M. Size-strain line-broadening analysis of anatase/brookite (TiO(2))-based nanocomposites with carbon (C): XRPD and Raman spectroscopic analysis. *Acta Crystallogr. B Struct. Sci. Cryst. Eng. Mater.* **2022**, *78* (Pt 2), 214-222.
